# Errors in Methods, Figure 1, Discussion, and Additional Contributions

**DOI:** 10.1001/jamanetworkopen.2019.7899

**Published:** 2019-06-21

In the Original Investigation titled “Comparative Survival Associated With Use of Targeted vs Nontargeted Therapy in Medicare Patients With Metastatic Renal Cell Carcinoma,”^1^ published June 14, 2019, there were errors in Methods, Figure 1, Discussion, and Additional Contributions. In the Statistical Analysis subsection of Methods, the second paragraph’s first sentence should read, “We used SEER-Medicare data to code sociodemographic characteristics (age, sex, race, marital status, census region, and urban vs rural residence), county-level characteristics (income, unemployment rate, and number of physicians per 1000 residents), and clinical characteristics (RCC grade, selected metastatic sites [lung, liver, bone, and brain], radiation status [yes or no], and nephrectomy status [yes or no]) for each patient.” The caption for Figure 1 should read, “Log-rank test comparing curves, *P* = .02 for overall survival and *P* = .001 for renal cell carcinoma–specific survival. n* indicates a cell size of less than 11, which is not reported per the National Cancer Institute data use agreement; NTT, nontargeted therapy; and TT, targeted therapy.” In the Limitations subsection of the Discussion, the text following the fifth sentence should read, “In addition, claims data may have variable accuracy for some clinical covariates we examined, such as metastatic site.^54^ Finally, rather than analyzing…”; the following has been included as reference 54: “National Cancer Institute. Measures that are limited or not available in the data. https://healthcaredelivery.cancer.gov/seermedicare/considerations/measures.html. Accessed June 6, 2019.” In Additional Contributions, the following should have been included: “This study used the linked SEER-Medicare database. The interpretation and reporting of these data are the sole responsibility of the authors. The authors acknowledge the efforts of the National Cancer Institute; the Office of Research, Development and Information, Centers for Medicare & Medicaid Services; Information Management Services (IMS), Inc; and the Surveillance, Epidemiology, and End Results (SEER) Program tumor registries in the creation of the SEER-Medicare database.” This article has been corrected.^1^
